# A Policy-Driven Large Scale Ecological Restoration: Quantifying Ecosystem Services Changes in the Loess Plateau of China

**DOI:** 10.1371/journal.pone.0031782

**Published:** 2012-02-16

**Authors:** Yihe Lü, Bojie Fu, Xiaoming Feng, Yuan Zeng, Yu Liu, Ruiying Chang, Ge Sun, Bingfang Wu

**Affiliations:** 1 State Key Laboratory of Urban and Regional Ecology, Research Center for Eco-Environmental Sciences, Chinese Academy of Sciences, Beijing, China; 2 Institute of Remote Sensing Applications, Chinese Academy of Sciences, Beijing, China; 3 USDA-Forest Service, Southern Research Station, Raleigh, North Carolina, United States of America; DOE Pacific Northwest National Laboratory, United States of America

## Abstract

As one of the key tools for regulating human-ecosystem relations, environmental conservation policies can promote ecological rehabilitation across a variety of spatiotemporal scales. However, quantifying the ecological effects of such policies at the regional level is difficult. A case study was conducted at the regional level in the ecologically vulnerable region of the Loess Plateau, China, through the use of several methods including the Universal Soil Loss Equation (USLE), hydrological modeling and multivariate analysis. An assessment of the changes over the period of 2000–2008 in four key ecosystem services was undertaken to determine the effects of the Chinese government's ecological rehabilitation initiatives implemented in 1999. These ecosystem services included water regulation, soil conservation, carbon sequestration and grain production. Significant conversions of farmland to woodland and grassland were found to have resulted in enhanced soil conservation and carbon sequestration, but decreased regional water yield under a warming and drying climate trend. The total grain production increased in spite of a significant decline in farmland acreage. These trends have been attributed to the strong socioeconomic incentives embedded in the ecological rehabilitation policy. Although some positive policy results have been achieved over the last decade, large uncertainty remains regarding long-term policy effects on the sustainability of ecological rehabilitation performance and ecosystem service enhancement. To reduce such uncertainty, this study calls for an adaptive management approach to regional ecological rehabilitation policy to be adopted, with a focus on the dynamic interactions between people and their environments in a changing world.

## Introduction

Ecosystem services are the benefits that people obtain from nature [1]. They are affected by a number of factors including changes in demographic, economic, sociopolitical, scientific and technological, cultural and religious, physical and biological conditions. The impacts of human activity on ecosystem services are most obviously reflected at the local and regional levels. Historically, natural, semi-natural, or managed ecosystems have been able to provide ecosystem services to meet the needs of social development. However, due to the accelerated growth of society, the gaps between the capacity of ecosystems to provide services and human needs are steadily widening. Over the last 50 years, 60% of worldwide ecosystem services have degraded due to increases in the global population and economic growth [2]. These human-ecosystem relationships have usually been governed by resource use and environmental conservation policies. However, policy issues have been under-evaluated in regards to their effects on improving ecosystem services and human-ecosystem relationships [3].

In China, widespread ecological degradation has constrained sustainable socioeconomic development in recent decades, particularly in the period before the end of 20^th^ century. For instance, 23% of the land area in China suffered ecological degradation of which approximately 35% of the Chinese population depended upon for ecosystem services between the early 1980s and 2000s. This also led to a reduced capacity for carbon sequestration during this period [4]. The estimated economic costs of interrelated problems associated with this degradation, including resource depletion, environmental pollution and ecological damage, have amounted to over 13% of the national Gross Domestic Product [5]. In recognizing the serious environmental and ecological issues during economic booms, the Chinese government implemented a series of policies towards ecological restoration. For example, the Grain to Green Program (GTGP) launched in 1999 is the largest land retirement program in the developing world and uses a public payment scheme that directly engages millions of rural households as core agents of project implementation. This is distinct from China's other soil and water conservation and forestry programs because it is one of the first, and certainly the most ambitious, “payment for ecosystem services” program in China [6]. During the 1999–2008 period, the Chinese Central Government made a direct investment of 191.8 billion RMB (approximately 28.8 billion USD) in the implementation of GTGP. This has resulted in the involvement of 0.12 billion farmers in retiring and re-vegetating 9.27 million hectares of sloping croplands [7].

**Figure 1:**
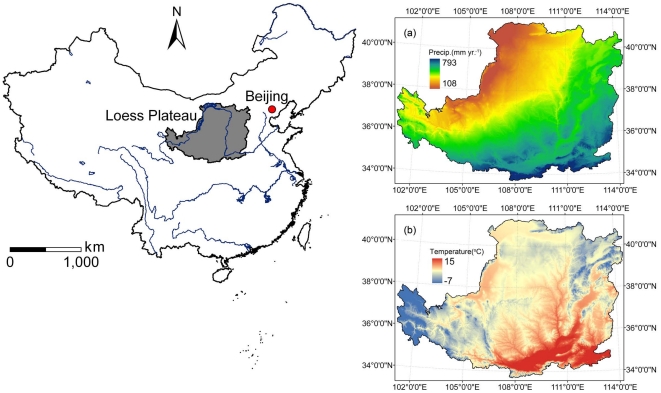
Location of the Loess Plateau and average climate conditions from 1999 to 2008. (a) Precipitation (b) Temperature.

This paper quantitatively evaluates the effects of GTGP implementation on ecosystem services in the Loess Plateau region (Figure 1), which is prioritized as a pilot region for the GTGP. It is necessary to assess the spatial and temporal changes in ecosystem services following the implementation of the GTGP in order to quantify the performance of large-scale ecological rehabilitation efforts and mainstream ecosystem services for future science-based decision-making [8]. The objectives of this study are to: a) examine the land cover change in the Loess Plateau between 2000 and 2008; b) quantify the changes in ecosystem services in terms of water regulation, soil conservation, carbon sequestration and grain production; and c) examine the socioeconomic effects of the GTGP and policy impacts on human-ecosystem relationships.

## Results

### Land cover change between 2000 and 2008 and the broad climate regime

**Figure 2:**
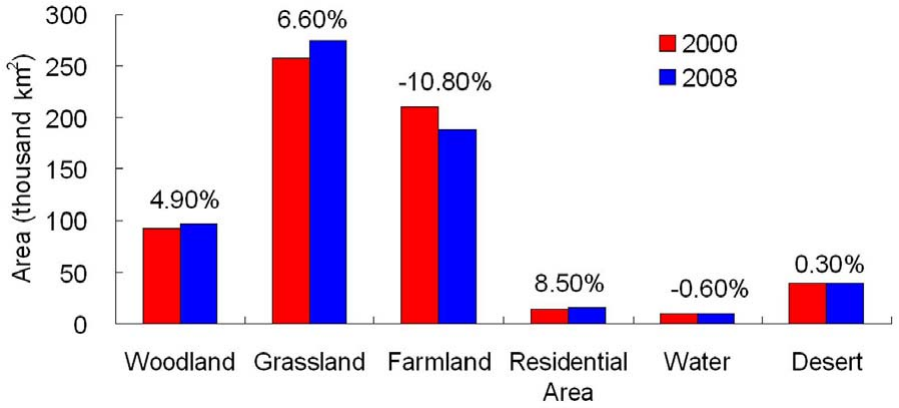
Coverage of each land cover type in the Loess Plateau, in 2000 and 2008. Numbers above bars indicate the change in area covered in 2008 as compared to 2000.

**Figure 3:**
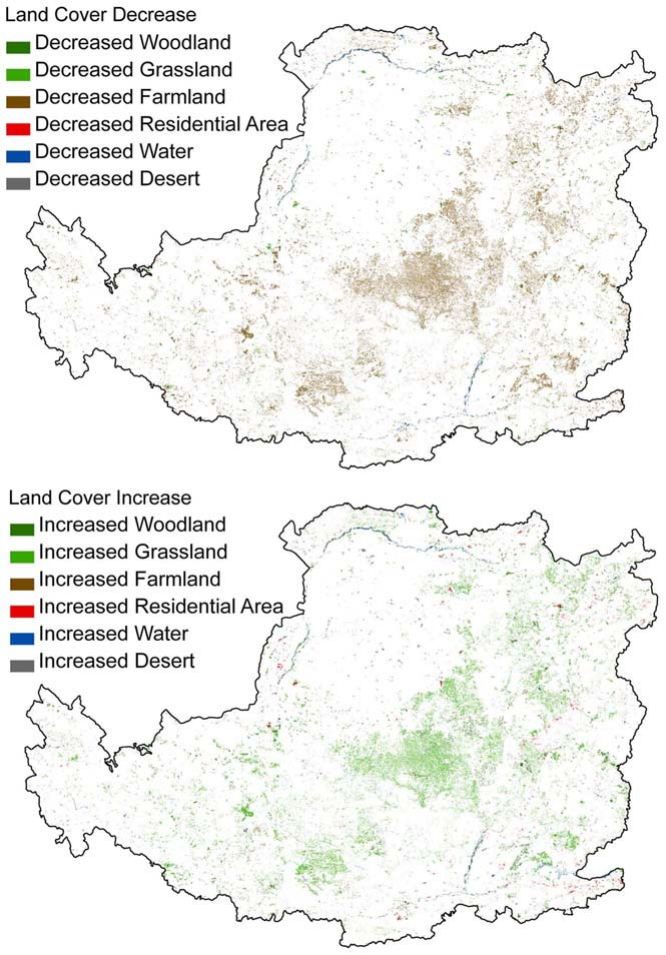
Decreased (above) and increased (below) land covers from 2000 to 2008.

Prior to the GTGP implementation, the Loess Plateau was dominated by grasslands and farmlands. Between 2000 and 2008 the land cover patterns of the Loess Plateau changed remarkably. Woodland, grassland and residential land cover increased by 4.9%, 6.6% and 8.5%, respectively. Farmland decreased by 10.8% and desertification increased slightly, by 0.3% (Figure 2). The increases in grassland and woodland were distributed along a northeast to southwest land strip (Figure 3) and were mostly converted from farmlands. This land cover change resulted in over 43% grassland, nearly 30% cropland, and about 16% woodland that dominated the Loess Plateau region in 2008.

**Figure 4:**
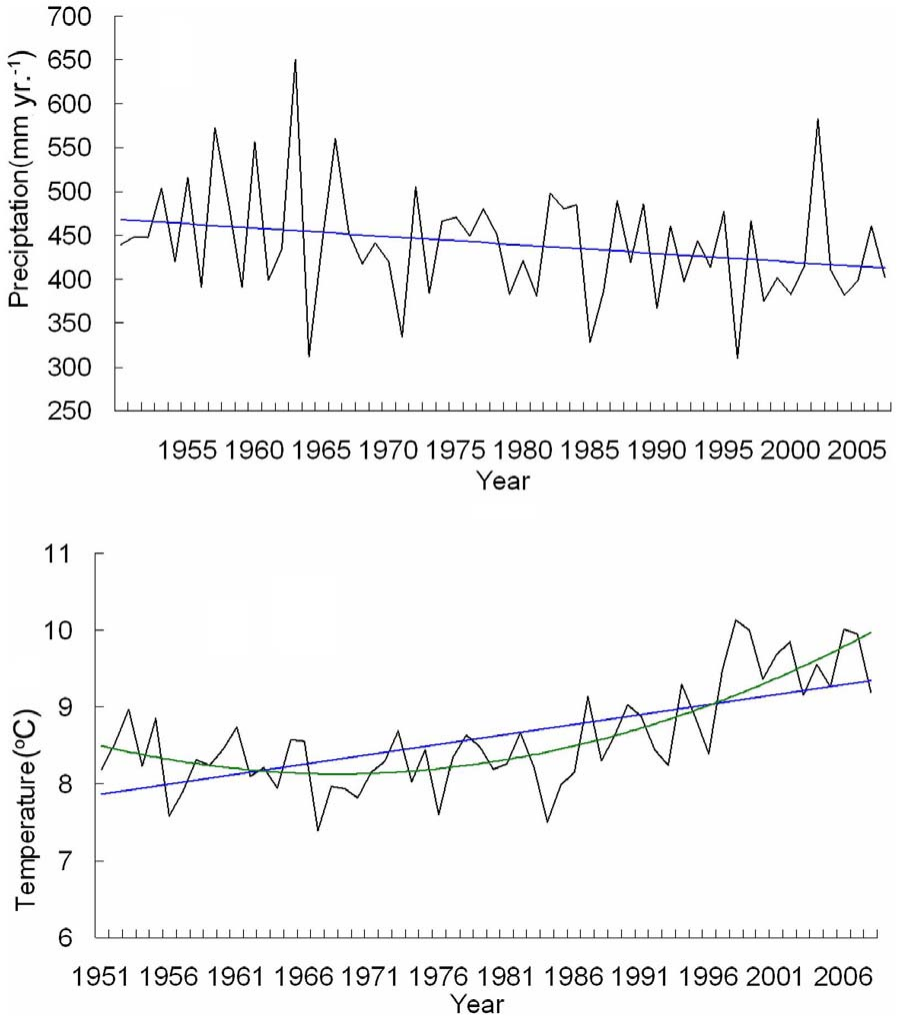
The trend towards a drier and warmer climate in the Loess Plateau region.

The regional climate condition of the Loess Plateau region has exhibited a warming and drying trend. This climate trend was revealed from the analysis of time series data between 1951 and 2008, obtained from 85 weather stations located in the Loess Plateau region (Figure 4). Precipitation was found to decrease annually by an average of 0.97 mm and temperature was found to increase annually by an average of 0.02°C.

### Hydrological regulation change

**Figure 5:**
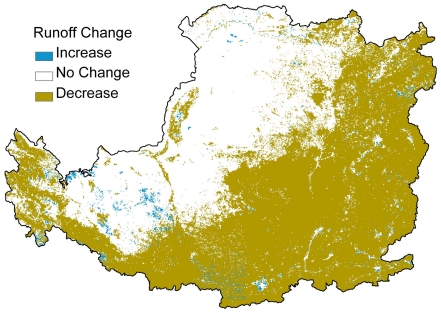
Average water yield change due to land cover change from 2000 to 2008.

Regional water yield decreased after the implementation of the GTGP. Over half of the study area (northeast to southwest of the Loess Plateau) experienced a decrease in runoff (2–37 mm/year) with an average 10.3 mm/year decrease in runoff across the whole Loess Plateau over the 2002–2008 period (Figure 5). While, water yield increased in some local areas which accounted for less than 10% of the Loess Plateau region.

### Soil conservation assessment

**Figure 6:**
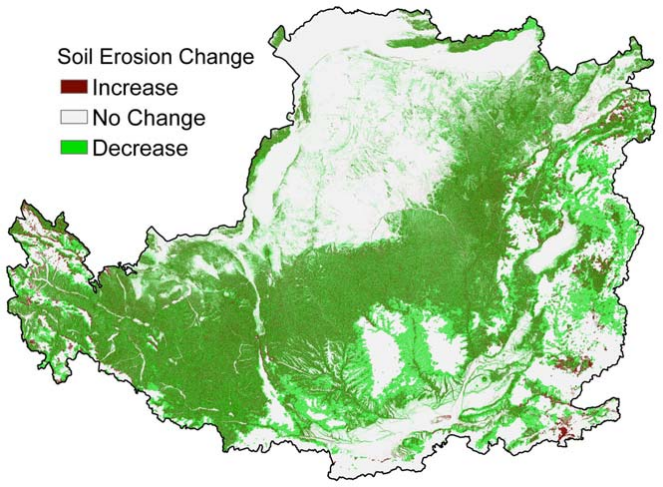
The change in soil erosion in the Loess Plateau region from 2000 to 2008.

Soil conservation in the Loess Plateau, represented as a decrease in soil erosion, has improved since 2000 as a result of vegetation restoration (Figure 6). The annual average soil retention of the study area between 2000 and 2008 was found to be 3.44 billion tons (Table 1), equivalent to an annual average soil retention rate of 63.3% [Soil Retention Rate (%) = 1.2603Time (years since 2000)+56.556, R^2^ = 0.3367 and P = 0.1. This linear relationship is not so significant statistically because of the large impacts from highly variable precipitations (Figure 4)]. The decreasing trend of soil loss per unit rainfall erosivity has also implied improvement on soil conservation service of the rehabilitated ecosystems (Table 1). After vegetation restoration, 84.4% of soil retention occurred on hill slopes with a slope angle between 8°–35°. However, the mean soil erosion rate in areas with a slope gradient of over 8° was still greater than 4,260 t km^−2^ yr^−1^ in 2008, which is far beyond the tolerable erosion rate of 1,000 t km^−2^ yr^−1^ [9]. Soil erosion is thus still considered one of the most critical environmental issues in the Loess Plateau and requiring further ecological rehabilitation efforts.

**Table 1:** Rainfall erosivity and soil retention characteristics in the Loess Plateau region from 2000 to 2008.

Year	2000	2001	2002	2003	2004	2005	2006	2007	2008
Rainfall erosivity [megajoules·mm/(ha·hour·yr)]	442.0	544.0	435.6	630. 8	487.8	408.7	456.3	539.0	434.4
Soil loss per unit rainfall erosivity (t)	0.048	0.044	0.044	0.040	0.046	0.045	0.038	0.031	0.035
Total soil retention(10^8^ t)	34. 5	30.8	26.1	49.8	27.7	33.4	31.9	48.6	26.9

### Carbon sequestration assessment

**Figure 7:**
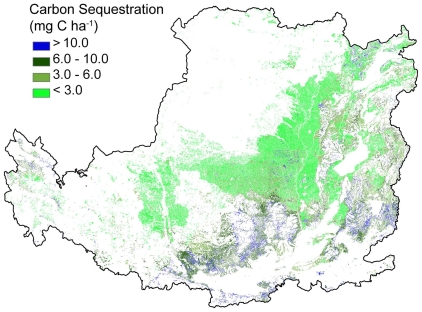
The spatial distribution of carbon sequestration.

Net carbon sequestration was estimated from vegetation and soil carbon change after re-vegetation was undertaken in 2000. The findings suggest that the ecological rehabilitation efforts have brought about significant positive impacts on carbon sequestration, with carbon levels in soil and rehabilitated vegetation found to be 11.54 Tg, and 23.76 Tg, respectively (Table 2). The spatial variation of carbon sequestration in the Loess Plateau is shown in Figure 7. The carbon sequestration is most evident from northeast to southwest including provinces of Shanxi, Shaanxi, Ningxia, and Qinghai, respectively.

**Table 2:** Area of cropland converted to forest (grassland) and the carbon sequestration by vegetation, soil and ecosystems in Loess Plateau between 2000 and 2008.

Types of conversion	Restoring to grassland	Restoring to shrub	Restoring to Broad-leaved forest	Restoring to coniferous forest	Total
Area of change (ha)	3.96×10^6^	4.85×10^5^	2.11×10^5^	1.73×10^5^	4.83×10^6^
Soil carbon storage (Tg)	8.25	1.81	0.72	0.77	11.54
Vegetation carbon storage (Tg)	7.16	11.30	3.24	2.06	23.76
Total (Tg C)	15.41	13.11	3.96	2.83	35.30

### Grain production

**Figure 8:**
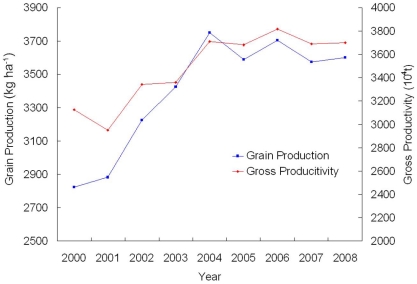
Grain and gross production change from 2000 to 2008 in the Loess Plateau region.

In the early stages of the GTGP implementation process (from 2000 to 2004), average grain productivity increased by approximately 1.3 times and then fluctuated around a productivity level of 3,614 kg/ha. As a result of this cropland productivity change, the gross grain production also increased by approximately 1.3 times between 2001 and 2006. The time and rate of the gross production change appeared to occur later and more slowly than the grain productivity change (Figure 8). Actual grain production increased across the whole of the Loess Plateau at a rate of 18% between 2000 and 2008.

## Discussion

This study's results suggest that GTGP has resulted in ecosystem property and service change under unfavorable climate change conditions. Specifically, the following changes have been detected: 1) Significant expansion of grassland, woodland and residential areas, and shrinkage of farmland; 2) Reduction in regional water yield; 3) Significant improvement in regional soil conservation capacity, grain production and carbon sequestration. Complex relationships may exist between these changes, as well as between the biophysical and socioeconomic conditions.

### Uncertainties involved in ecosystem service assessment

Several factors affected the accuracy of estimating annual water yield at the regional scale. Firstly, the complex terrain of the Loess Plateau presented a challenge for deriving the spatial distribution of annual precipitation that was interpolated from climate records at 172 weather stations in the Loess Plateau region. In addition, the seasonal and inter-annual variability of precipitation was considered to be high. The large spatial and temporal variability in precipitation thus made accurate mapping of precipitation distribution difficult at the 1-km resolution. Although the evapotranspiration (ET) modeling results were believed to be much closer to reality than the results obtained from the remote sensing based product (MODIS-ET), uncertainty remained over the seasonal distribution of ET by land cover type. Uncertainty also surrounded monthly ET estimates for two reasons: 1) Change in water storage may not have been negligible for certain wet years; and 2) Water resource use by communities and the impacts of soil conservation structures (e.g., check dams), were not considered. Anyway, water yield estimation is still an inaccurate science at this point in time [10], particularly at larger spatial scales. Overall, the method used in this paper may introduce systematic errors at a level of approximately 15% [11].

The estimation of soil conservation was undertaken through the application of the Universal Soil Loss Equation (USLE) together with remote sensing. The USLE is based on a statistical relationship established from a large number of plot scale rainfall-erosion experiments [12]–[13]. It estimates rill and inter-rill soil detachments on hill slopes from rainfall, soil and soil cover parameters, and management factors [14]. Therefore, it is a suitable method to estimate the effect of hill slope vegetation rehabilitation on soil conservation. However, this effect may have been overestimated in this research due to the omission of the local sediment deposition process [13]. Overestimation may have also occurred due to setting the control for soil conservation effects to a scenario of no vegetation cover or erosion control practice. Overestimation is evident after comparing soil conservation results to those from another similar soil conservation assessment using different methods, which reported an average soil conservation rate linked to vegetation restoration of 38.8% in the Zuli River basin of the Loess Plateau region [15]. These overestimations were made for the absolute values of spatial explicit annual soil conservation measurements but did not exclude the soundness of comparisons between annual soil conservation services brought about by vegetation rehabilitation. Uncertainties were also identified from the estimation of input parameters for the USLE [14]. Therefore, parameters established and experimentally verified in the Loess Plateau region were used for estimating the different factors in the USLE [16]–[19] to reduce this source of uncertainty.

For the assessment of carbon sequestration, only the effects on areas with land cover transitions from farmland to forest, shrub, or grass were considered. However, evidence from the Loess Plateau suggests that significant carbon sequestration effects could also be detected in grassland and forestland from the process of ecological succession [20]. This research may therefore underestimate the carbon sequestration effects at the regional scale due to the exclusion of carbon sequestration effects associated with grassland, shrubland, and forest ecosystems that existed before implementation of the GTGP. Soil carbon sequestration effects at the sample point scale were also estimated for equal soil depths (20 cm) because of insufficient soil bulk density data. Furthermore, regional soil carbon sequestration effects were estimated through the upscaling of 103 samples collected from the Loess Plateau using a multi-regression method. The accuracy of the results from the CASA (Carnegie-Ames-Stanford Approach) model, which is fundamental to vegetation carbon sequestration assessment, is largely dependent on the resolution of remote sensing data. MODIS data at 1-km resolution and parameters transferred from national scale studies [21]–[22] were used for this research. All the above methods can introduce errors or uncertainties in the estimation of vegetation and soil carbon sequestration. These errors or uncertainties can be reduced with more soil data, higher resolution remote sensing data and localized model parameters. Given these uncertainties, the major characteristic of the carbon sequestration effects of the GTGP was revealed to be the dominance of vegetation carbon accumulation, which was found to be approximately twice the level of soil carbon sequestration in this study. This figure was 2.3 (vegetation carbon accumulation divided by soil carbon sequestration) in similar research conducted in Yunnan province of southwestern China [23].

### Synergies and tradeoff between ecosystem services

The implementation of a large-scale vegetation rehabilitation program under a regional warming and drying climate (Figure 4) may contribute to the decrease of stream flow in the Loess Plateau region [24] because of the potential increase in vegetative water consumption. Vegetation cover in the Loess Plateau region has expanded due to a significant increase in grassland and woodland areas (Figure 3). The amount of vegetation cover improvement achieved is higher than the national scale target of the GTGP to increase grassland and woodland coverage at a rate of 4.5%. This result was also supported by other research which estimated that vegetation cover in the whole Loess Plateau increased at an approximate rate of 6–8% during 2000–2006 [25] or 12.5% at local level in the central Loess Plateau during 1998–2005 [26]. The net primary production of regional ecosystems in the Loess Plateau that experienced a significant increase or remained stable between 1999 and 2008 accounted for 65.8% and 14.3% of the region, respectively [27]. Consequently, the trend of improving carbon sequestration, soil conservation and grain production may indicate that these key ecosystem services act in synergy. The decrease in regional water yield and the improvement in vegetation cover may be considered a tradeoff, as water resources and vegetation typically maintain an inverse relationship in semi-arid water limited environments under given climate conditions. However, both elements contribute significantly to the enhancement of soil conservation and carbon sequestration (Figure 6–7 and Table 1). Due to the decline of regional water yield, nitrogen (influenced by population pressure and fertilizer use) and phosphorus (sourced from soil erosion in the Loess Plateau) transported in the lower reaches of the Yellow River have reduced significantly since the late 1990s [28]. The implementation of the GTGP vegetation rehabilitation program may therefore improve the water quality of the middle and lower reaches of the Yellow River, however, water shortage issues [29] may potentially be exacerbated. The significant improvement in cropland productivity were attributed to factors such as agricultural technological growth, the construction of high quality basic croplands (e.g., terrace croplands and check-dam derived croplands), the increase in resource input and farming management, and the improvement of extension services [30]–[31] as complementary or insurance measures for ecological rehabilitation.

### Grain to Green Program and local empowerment

Under the GTGP, the government offered grain and cash to farmers annually as compensation (grain subsidy of 1500 kg/ha plus cash subsidy of RMB 300/ha) for their opportunity costs in discontinuing farming on sloping croplands [32]. The program has helped numerous farmers to gradually change their income structure by shifting from grain production to other income-generating activities [33]. Subsequently, the employment and sources of family income of farmers have been diversified due to the economic compensation obtained through the GTGP, which ranges from 10% to 30% of their total income [34]–[35].

The rural economic capacity of the Loess Plateau has also improved at both the regional and farmer household levels. Data from the National Bureau of Statistics of China indicates that the net per capita income of farmers in the Loess Plateau region increased annually from 1998 to 2007 at a rate of 8.6%, which could be actually reduced to 4.5% after subtracting the annual average inflation rate of 2.1% and the rural consumption price increasing rate of 2% in China during 2000–2008. The ratio of farmer respondents reporting significant increases in household income after the implementation of the GTGP varied with different study sites and ranged from 55% to over 90% [34], [36]. During the implementation of the GTGP, local farmers developed a greater understanding of and support for ecological restoration programs [37]. However, the direct economic compensation from the GTGP has only been a minor contributor to farmers' income. The more significant effect of the GTGP has been to accelerate the socioeconomic transition from a food production-based rural community to a more active and profitable labor migration dominant rural economy (i.e. where the rural labor force can migrate to urban areas to earn a living or run a business).

### Sustainability through adaptive management

This study suggests that the ecological rehabilitation policies of the GTGP and associated soil and water conservation measures implemented in the Loess Plateau tended to facilitate synergies on carbon sequestration, soil conservation, grain production, and farmers' economic welfare. These synergies are important goals that ecosystem management tries to reach.

The successful performance of ecological rehabilitation programs discussed above was largely due to the innovative ecosystem management systems and mechanisms. Close cooperation between local government and other stakeholders was found to be important for capitalizing on synergies between ecological rehabilitation initiatives, and for maximizing the outcomes of ecological management activities [38]. External funding other than government sources, such as private sectors, enterprises, and the World Bank, were also important in the success of restoration programs [39]–[40]. Project selections and designs have been increasingly informed by feasibility studies and demonstrations. Project planning has been taking a preliminary adaptive approach, informed by ongoing monitoring and evaluation as well as performance assessment [39], [41].

Quantitative assessments of present ecological restoration policies have been increasingly available and the sustainability issues of regional ecological restoration programs have been recognized. For example, when non-native tree species were planted at a high density, soil drying was observed during re-vegetation which undermines the long-term capacity of soil to sustain ecosystems under a semi-arid climate [42]. Soil drying is at least partly due to the ecological rehabilitation policy that gives more weight to planting trees and less consideration of natural restoration that is more tailored to the local environment [43].

From a socioeconomic point of view, the sustainability of ecological rehabilitation depends largely on the economic incentives or benefits produced by the implementation of such activities. As the GTGP has been implemented in over 200 counties across seven provinces in the region, data insufficiency and uncertainty excluded a cost benefit analysis of the GTGP across the whole Loess Plateau. A local scale analysis in Dunhua county indicated that the net benefit (sometimes negative) varied widely according to geographical location (or environmental context), land productivity and discount rate [44]. Subsequently, the risk of re-cultivation of re-vegetated croplands will remain high if the policy-related economic compensation measures from the government are terminated [37], [43], [45].

Therefore, to improve the actual performance of regional ecological rehabilitation efforts, an adaptive management paradigm needs to be established to integrate the government-motivated “top-down” approach and the local stakeholder motivated “bottom-up” approach, with balanced considerations of the dynamics and sustainability requirements of the targeted ecological-socioeconomic coupled systems. Ecological rehabilitation is widely used in reversing environmental degradation and can contribute to the improvement of ecosystem services and adaptability to climate change [46]–[47]. The success and sustainability of ecological rehabilitation efforts depend on the scientific understanding of the interactions between people and their surrounding ecosystems, rather than merely the ecosystems themselves [48]. Consequently, the way to secure sustainability in ecological rehabilitation and ecosystem service enhancement is to ensure net benefits, or at least, no net loss to the stakeholders and ecosystems involved in the adaptive management framework. An adaptive management approach allows for flexibility in human and financial resource allocation, an expansion of knowledge on the dynamic socioeconomic-ecological coupled systems, and efficacy of management operations [49]. The experience of ecological rehabilitation and the change in key ecosystem services in the Loess Plateau region exemplified the positive effects of environmental policies and the necessity of adopting an adaptive management approach.

## Materials and Methods

### Study area description

The Loess Plateau region is located in the middle reaches of the Yellow River basin in Northern China (Figure 1) and experiences arid and semi-arid climate condition over an area greater than 600,000 km^2^. Precipitation occurs between June and September and accounts for 60–70% of the annual total in the form of high intensity rainstorms. The Loess Plateau is an ecologically vulnerable region and is well known for its high soil erosion rates and heavy sediment loads. The average erosion modulus is 5,000–10,000 t/km^2^, with the highest rate up to 20,000–30,000 t/km^2^ [9]. The areas characterized by slopes of 8–35 degrees are the main source areas for soil erosion and represent 45.63% of the whole Loess Plateau region. Therefore, restoring vegetation in these areas will play a key role in mitigating soil erosion.

The Loess Plateau comprises 6.67% of the territory in China and supports 8.5% of the Chinese population. By the end of 2007 the human population in the Loess Plateau region reached a magnitude of approximately 0.114 billion and a population density of 168 persons per square kilometer, a number four times that of the early 1910s. As a result, human pressure upon land resources has increased significantly in this region. Soil erosion has been accelerated by intensive land use (e.g., slope farming) and exploitive management for thousands of years, resulting in the loss of grassland and natural forest. Due to its great geographical magnitude, the Loess Plateau has diverse habitat conditions for different vegetation types which have shifted historically because of climate change. It can be inferred from literature that grassland and forest steppe were the dominant vegetation types across the whole Loess Plateau region. Forest was also dominant at a local scale in mountainous and valley areas in the Quaternary and particularly the Holocene periods [50]–[53]. In the last 2000 years, the vegetation in the Loess Plateau region has experienced significant degradation due to increasingly intensive human activities [52], [54]. In 2000, woods (i.e. forests and shrubs) and grasses in the Loess Plateau Region covered areas of 77.3 and 252.8 thousand square kilometers, respectively (Figure 2). At present, the forest area in the Loess Plateau region accounts for only 7% of the total forest area in China [55].

### Land cover change

Landsat TM/ETM images from 2000 were used to extract land cover data for the Loess Plateau. Prior to image interpretation, remote sensing data was geo-referenced through the use of 1∶100,000 topographic maps. For each Landsat TM/ETM image a minimum of 30 evenly distributed sites were selected as Ground Control Points (GCPs). The Root Mean Squared Error of geometric rectification was less than 1 pixel (or 30 m). Land cover types were identified using ArcMap and based on the spectral reflectance and structure of objects. A total of 27 land cover subtypes in the study area were further grouped into six aggregated land cover types: woodland, grassland, farmland, residential areas, water bodies and desert. Based on the land cover map from 2000, the land cover classification of the Loess Plateau from 2008 was updated using China-Brazil Earth Resources Satellite (CBERS-2b) images. These images have a 20 m ground resolution and a similar amount of spectral bands as Landsat ETM images. To support image interpretation and validate the land cover map from 2008, a field survey was conducted to evaluate the classification accuracy. Field-measured land cover types and photos located with GPS coordinates were collected across the whole study area. Classification accuracy was measured as 95% at the level of the six aggregated land cover types.

### Hydrological regulation

Water yield was used as an indicator of hydrological regulation. Water yield at the watershed-scale was modeled as precipitation minus evapotranspiration (ET), based on the assumption of negligible water storage change in the Loess Plateau region on an annual time scale. Monthly ET (mm) was estimated by ET = 9.78+0.0072*PET*PPT+0.051*PPT*LAI, where PET represents potential evapotranspiration (mm), PPT represents precipitation (mm), and LAI represents leaf area index (dimensionless) [11]. PET (mm) was calculated using the Hamon method [56]. The climatic parameters were obtained from the National Climatic Bureau and interpolated with ANUSPLIN [57]. LAI was derived from SPOT VEGETATION NDVI based on the relationship between NDVI and LAI for different types of land cover [58]. The monthly Loess Plateau ET model was calibrated and validated using runoff data from 46 basins in the region. This runoff data was retrieved from the web-based hydrological and sediment database of the Loess Plateau (http://www.loess.csdb.cn/hyd/user/index.jsp). The structure of the above monthly ET equation follows the empirical relationships established between monthly ET and the main influencing factors of 13 ecosystems with wide geographic distribution [11]. The present form of the ET equation has been established since calibration and validation was undertaken and is suitable for use in the Loess Plateau region.

### Soil conservation

The soil conservation services of re-vegetation have been measured since 2000 by calculating the decrease in regional soil loss or regional soil retention on hill slopes. Soil retention is calculated as soil loss without vegetation cover and soil erosion control practices minus that under the current land use/land cover patterns and soil erosion control practices. The Universal Soil Loss Equation (USLE) is the most widely used method for soil erosion modeling and assessment [59] and was applied to quantify the amount of annual soil loss for the two situations described above. Soil retention can be expressed mathematically as: 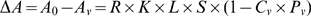, where 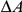 is the amount of soil conservation (t·ha^−1^·yr^−1^); 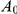 is the potential soil erosion without vegetation cover (t·ha^−1^·yr^−1^); and 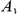 is the soil erosion under current land cover and management condition(t·ha^−1^·yr^−1^). *R*, *K*, *L*, and *S* represent rainfall erosivity [megajoules·mm/(ha·hour·yr)], soil erodibility [t ·ha· h/(ha·megajoules· mm)], slope length, and slope angle factors respectively. 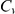 and 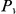 refer to current vegetation cover factors and erosion control practice factors, respectively. *L*, *S*, 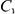, and 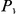 are all dimensionless factors.

### Carbon sequestration

Soil Organic Carbon (SOC) sequestration for the GTGP in Loess Plateau was estimated by using a multiple regression approach. This approach included precipitation, the vegetation types converted from sloping croplands, and the time duration after conversion as the main independent variables. In the GTGP, sloping croplands (generally with a slope >15°) were converted principally into grassland, shrub, broad-leaved forest and coniferous forest. SOC sequestration for the GTGP in the Loess Plateau was estimated based on these four established ecosystems in two major climate zones, defined as zones with precipitation less than (north Loess Plateau) and greater than (south Loess Plateau) 550 mm. In each of the two climate zones, SOC sequestration under each plantation type was calculated by using the SOC sequestration rate derived from a multiple regression and the area of cropland involved in this plantation type. The total SOC sequestration across the whole Loess Plateau was determined from the sum of the SOC sequestration estimated in the four plantation type in the two climate zones. The multiple regression was undertaken based on SOC sequestration data collected from the top 20 cm soil layer collected from 103 samples across the Loess Plateau [ log(*Y*) = 2.648–0.366 *P*- *α*×*U*+0.023 *y* (R^2^ = 0.256, P<0.05, N = 103). *Y* is the SOC sequestration rate (Mg C/ha) and *P* is a dummy variable representing precipitation. The value of *P* is set at 0 when precipitation is above 550 mm, while *P* is set at 1 when the precipitation is below 550 mm. *U* is also a dummy variable representing land use change. When cropland was converted into grassland, shrub, broad-leaved forest and coniferous forest, the value of *α* was set at 0.727, 0.533, 0.633 and 0, respectively. *y* is a variable representing plantation age]. Carbon sequestration in the vegetation of each plantation type was estimated from the carbon sink efficiency of the vegetation type and the NPP was calculated by using the CASA (Carnegie-Ames-Stanford Approach) model [21] [CSE = Cseq/NPP×100), where CSE is carbon sink efficiency; Cseq is the carbon sequestration in vegetation (MgC/ha/a); and NPP is Net Primary Productivity (gC/m^2^/a)]. The value of CSE of grassland, shrub and forest (inclusive of broad-leaved forest and coniferous forest) was set at 0.015, 0.036 and 0.057, respectively according to Fang et al. [22].

### Grain production

Data on grain production was obtained from provincial level Bureaus of Statistics in the Loess Plateau (287 counties in seven provinces).
